# Social and Poor-Law Problems

**Published:** 1906-06-02

**Authors:** 


### SOCIAL AND POOR LAW PROBLEMS.

### THE PRISON REST CURE.

Australia is beginning to awake to the folly of one kind of protection—that of forbidding the sale of prison-made products in the open market. The Melbourne *Age* has recently published a satirical article dealing with the so-called "hard labour" at Pentridge, one of the Government convict establishments. Hard labour is the name given to a kind of dawdling through the time, which, if it punishes the criminal at all, can do so only by causing him infinite *ennui*. Even in the stoneyard the convicts proceed with their work, according to a journalist visitor, as if they were making a geological inspection of the road-metal supplied. One man occasionally chips a fragment of the stone before him, while half a dozen watch the process or survey the sunlit landscape. At the farm, some of the men "contemplate their shovels and painfully thrust them into the earth at long intervals. Others sleepily watch the maize and vegetables growing as if there were imminent danger of the plants running away to seed before anyone could raise a hand to stop them." In the mat-making and brush-making yards, in every department of prison industry, the tale was the same—the less the convicts did the better the officials were pleased. Not that they desired to encourage idleness, but that they had no use for the product when the work was finished. They were allowed to give the goods made in the prison to other Government departments, but not to sell them to the public, for fear of competing with free labour. Before the regulations forbidding the selling of prison-made goods in the open market were promulgated, the output of Pentridge was far greater. In 1897-1898 there were sold 156 cricket carpets made at the prison, and the next year 137. Last year only 15 were sold. The foreman of the mat-making shop says that where formerly there were 90 to 100 prisoners working there are now 30 pottering. The men certainly learn a trade there, and no one would desire to do away with any educational effect that prison training may have, but the opinion of the foremen who instruct the prisoners is that they seldom or never put in practice, when they return to freedom, the trade they learned in captivity. And indeed one may doubt if the dawdling ways they learn in prison will earn them an honest living outside. They are far from maintaining themselves where they are. At present each of these convicts costs the Colony £43 11s. 4d. annually, and to this expense he contributes by his labour only £4 8s. 2d. A life which, professing to inflict on a man "hard labour," enables him only to earn such a paltry sum as this, can hardly be described as punitive.

Our newspapers are not so frank about the real meaning of "hard labour" as the Melbourne *Age*, but even here it is more than suspected that prison labour is uncommonly akin to idleness. Trades are taught, but not as a rule by men who have ever earned their living at the avocation they teach, but by warders who have taken a more or less amateurish education in it. The work has no definite aim, beyond teaching the prisoners, who are not particularly anxious to be taught, though they may be willing to enter one of the workshops because of the chances of conversing with their fellows through being engaged in a joint task, and the good marks they may earn by taking up a craft. But the work has no market value, and because nobody wants it, turnkey-teacher and prisoner-pupil alike are indifferent to its progress. Who would make haste to build a wall which he knew was to be immediately knocked down again, and rebuilt anew as a lesson in bricklaying? Nothing paralyses energy so much as the knowledge that its results are futile. If convicts do not learn to find an interest in work, to realise that there is something worth doing for even such as they, the moral effect of their training will be *nil*, and that being so, it matters little what trade they are taught: they will never pursue it seriously. And to tell the truth, no outside master would keep on such workmen for a week.

But if ever a protest against this expensive futility is made, there arises the cry even here, and still more insistently in Australia, of allowing prison labour to compete with free labour. The attention which this cry deserves depends a good deal on what is meant by competition. It would be unjust to undersell the free workman, who must maintain himself and his family, by offering goods of equal quality, made by a worker who (for his sins) is being maintained by the State, at a lower price. But if the articles, being equally good, are sold only at the fair market price—as settled by the ordinary laws of supply and demand—does the free labourer suffer any wrong? He must face competitors, and what does it matter to him where his competitors live and work—in a prison or in another town? The argument that the work done by a prisoner might be done by a free man belongs to the narrowest school of tradesunionism, to the spirit that ruined the medieval guilds, that which would charge prohibitive prices for the product in order to enrich the producer, with the sure result of limiting the production altogether, and in the end killing the trade. The workman who objects to prison labour on those grounds is simply asking the State to maintain in idleness a possible competitor, and might as well ask the Government to keep any one of his mates idle in order to leave the work for him. So long as prison-made goods are not sold at less than ordinary rates there is no undue competition, nor is it likely that the amount of prison-made goods put upon the market would ever seriously affect prices unless they were wilfully sold at a loss.

If such a use of prison labour does not meet with general approval, there remains only that the Government should extend its field of operations so as to give more active and profitable employment to its compulsory guests. There are strong objections to the hiring out of convict labour, as it would be difficult to secure effective guarantees against tyranny, and even cruelty, on the part of the contractors who hired the men. But all the tasks which the Government is asked to provide for the unemployed would be suitable for occupying convict labour. If such work as afforestation, reclaiming of foreshores, road-making, and the like really are needed, why not set convicts to do it? If there is no such need there is no reason for getting it up for the unemployed. One can conceive of a country where most public buildings would be erected by convict labour, and where the clothing needed for the soldiers and police was woven and made in prisons. Of course we should have the old cry of interfering with free labour. But the taxpayer would probably feel that there was something to be said for getting some return out of those whom he was obliged to keep; and the philanthropist would see some merit in a plan which prevented punitive "hard labour" being a synonym for idleness, and acted as a real deterrent to crime, besides sending men out when their term of imprisonment was up with some capacity, and perhaps some liking, for the labour by which honest men earn their bread.

